# What is the quickest scoring system to predict percutaneous nephrolithotomy outcomes? A comparative study among S.T.O.N.E score, Guy's Stone Ccore and CROES nomogram

**DOI:** 10.1590/S1677-5538.IBJU.2016.0586

**Published:** 2017

**Authors:** Fabio C. Vicentini, Felipe R. Serzedello, Kay Thomas, Giovanni S. Marchini, Fabio C. M. Torricelli, Miguel Srougi, Eduardo Mazzucchi

**Affiliations:** 1 Seção de Endourologia, Departamento de Urologia, Hospital de Clínicas, Faculdade de Medicina da Universidade de São Paulo, SP, Brasil; 2 Faculdade de Medicina da Universidade de São Paulo, São Paulo, SP, Brasil; 3 Stone Unit, Guy's and St. Thomas' National Health services foundation Hospital, London, United Kingdom

**Keywords:** Nomograms, Diagnosis, Calculi

## Abstract

**Objective:**

To compare the application time and the capacity of the nomograms to predict the success of Guy's Stone Score (GSS), S.T.O.N.E. Nephrolithometry (STONE) and Clinical Research Office of the Endourological Society nephrolithometric nomogram (CROES) of percutaneous nephrolithotomy (PCNL), evaluating the most efficient one for clinical use.

**Materials and Methods:**

We studied 48 patients who underwent PCNL by the same surgeon between 2010 and 2011. We calculated GSS, STONE and CROES based on pre-operative non-contrast computed tomography (CT) images and clinical data. A single observer, blinded to the outcomes, reviewed all images and assigned scores. We compared the application time of each nomogram. We used an analysis of variance for repeated measures and multiple comparisons by the Tukey test. We compared the area under the ROC curve (AUC) of the three nomograms two by two to determine the most predictive scoring system.

**Results:**

The immediate success rate was 66.7% and complications occurred in 16.7% of cases. The average operative time was 122 minutes. Mean application time was significantly lower for the GSS (27.5 seconds) when compared to 300.6 seconds for STONE and 213.4 seconds for CROES (p<0.001). There was no significant difference among the GSS (AUC=0.653), STONE (AUC=0.563) and CROES (AUC=0.641) in the ability to predict immediate success of PCNL.

**Conclusions:**

All three nomograms showed similar ability to predict success of PCNL, however the GSS was the quickest to be applied, what is an important issue for routine clinical use when counseling patients who are candidates to PCNL.

## INTRODUCTION

Nephrolithiasis is a common condition, with high prevalence and recurrence, constituting one of the most common diseases of the urinary tract (1). The disease affects 5% to 15% of the world population, with a peak incidence in young adults between the third and fourth decade of life (2, 3). The surgical treatment of nephrolithiasis has advanced substantially in recent years. Percutaneous nephrolithotomy (PCNL) remains the gold standard modality for treatment of complex renal stone and/or high volume stone (4–6). Despite the establishment of PCNL as one of the most important methods for the treatment of kidney stones, currently there is no gold standard tool for predicting success and complications associated with this surgery (7). This is important because a scoring system could help the surgeon in planning surgical strategies, predict the success rate and complications, result in better patient counseling, and facilitate comparison of outcome between the different institutions (7).

There are a few scoring systems in the literature which assess pre-operative parameters and predict the success rate of PCNL (8–11). They have showed to positively correlate with outcomes or complications, but comparison among them is required in order to determine the most practical and applicable one in clinical practice. Currently, three nomograms have been more extensively studied: Guy's Stone Score (GSS) (8), the S.T.O.N.E. Nephrolithometry (STONE) (10) and the nomogram of the Clinical Research Office of the Endourological Society (CROES) (9). The GSS consists of a nomogram using as parameters the amount of stones, their renal location, history of spina bifida or spine injury, and the association with possible anatomical changes, as horseshoe kidney: nephrolithiasis burden is classified in 4 degrees related to different success rates in PCNL (8). The parameters used in the STONE include stone size, distance to the skin, the degree of obstruction in the urinary tract, the number of renal calices involved, and stone density (10). Finally, the CROES uses variables such as area, number and location of the stones, previous treatment, staghorn stone and number of cases treated per year in the institution (9).

Some studies have shown that all nomograms correlated well with success or complications (12–18) and that they have similar ability to predict surgical outcomes (19–22). Nevertheless, there are no studies assessing these nomograms in clinical practice, where time is an important factor. In this study, we aim to compare the acquisition times for the most used nomograms.

## OBJECTIVE

Our primary goal was to compare the application time of GSS, STONE and CROES, evaluating which one is quickest to be applied in clinical practice.

Our secondary objective was to compare the ability of the nomograms to predict the immediate success rate of PCNL.

## MATERIALS AND METHODS

We performed a retrospective review of medical record data from a prospectively collected database. In our institution, we perform >180 PCNL/year. We analyzed patients who underwent PCNL between February, 2010 and December, 2011 at our institution by the same senior urologist (FCV) under the same technique, as previously described (22). Briefly, under general anesthesia, all patients were positioned in the supine decubitus with the posterior axillary line located just outside the border of the surgical Table; the flank was extended to increase the space between the last rib and the iliac crest; all csPCNLs were performed without boosters under the flank and all patients were maintained in the same position during the entire procedure.

Tract dilation was performed with fascial dilators (numbers 10, 20 and 30Fr, sequentially) and the Amplatz^®^ sheath was placed. Nephroscopy was performed with a 26Fr rigid nephroscope (Karl Storz, Munich, Germany) and we used an ultrasonic lithotripter for stone fragmentation and suction. The stone free status was verified with combined fluoroscopy and flexible nephroscopy. A 16Fr nephrostomy tube was placed at the end of the procedure in case of bleeding, residual stones, solitary kidney, suspected pelvic injury, or multiple tracts. A 6Fr ureteral catheter was routinely placed; in cases of ureteropelvic junction significant edema, extensive pelvic injury, or ureteral manipulation, a double-J stent was used instead. Operative time was considered from the beginning of cystoscopy for ureteral catheter insertion to the end of nephrostomy placement.

Complication was defined as any deviation from normality in the peri or postoperative period of 72 hours, using the Clavien Scale, validated for postoperative complications in PCNL (23). Complication rate comparison was not one of our objectives due to the fact that GSS was not developed for this purpose. The study protocol had ethical review board approval.

### Selection criteria

Exclusion criteria comprised patients younger than 18 years old and older than 70 years old, patients with inadequate analysis by preoperative CT (low resolution or not performed in our service) and without at least one follow-up consultation in a 60 days period.

### Measurements

We calculate the GSS, STONE and CROES of all patients based on preoperative CT images as described by Thomas et al.(8), Okhunov et al. and Smith et al.(9), respectively. A single observer (FRS) reviewed all images and performed scoring according to each system. The reviewer was a 5^th^ year medical student (of a total of 6 years), with basic knowledge in radiology, with no previous use of any of the scoring systems, who was initially trained to evaluate non-contrast CT scans by calculating the three scores for 20 different cases under supervision of two senior urologists before initiating the study. We did not use these cases for our study, just for training the observer. A concordance index among the calculations of the 3 observers for these 20 cases was 0.86, showing that the reviewer has been properly trained. We analyzed all the images on the computer screen and all parameters were acquired through the image display program. The high concordance index allowed us to make all the analysis based on the data calculated by only one observer. The observer also had a Table with the data regarding physical examination, history of previous surgery and presence of spina bifida or not. The time for clinical data analysis was not considered, because in a clinical setting this information usually is already known by the assistant when analyzing the CT images, according to a regular patient evaluation.

### Acquisition Time

The time required for application of nomograms was individualized for each nomogram in each case. For this, we used a simple timer. The count began at the time the researcher started the evaluation of the imaging exam and ended when the investigator obtained the final score of the nomogram. First, we calculated the GSS consecutively for all patients and the data inserted into an Excel Table (Microsoft, California). Then, in another day, we calculated the STONE parameters also for all patients. Stone burden was estimated in mm^2^ using the formula, Σ (0.785 x length_max_ x width_max_) and the value was automatically generated by the software (24). Finally, again in another day, we obtained the CROES from the same images.

We did not reuse the results of measurements taken for one nomogram for calculating the other one and we did all the measurements in different days in order to guarantee that the observer did not have memory of the image previously seen. Every nomogram had its proper Excel Table, with the patient's demographic data, history of previous surgery and presence of spina bifida or not.

### Definition of success

We defined success as stone fragments ≤4mm on CT scan on the first postoperative day (POD1). Stone-free rate refers to no identification of any stone fragment on the POD1 CT. Final success rate was defined as the result of the last radiological exam performed after all the auxiliary procedures, consisting of revision PCNL, external shockwave lithotripsy or flexible nephroscopy.

#### Statistical analysis

We calculate the sample size based on an expecteddifference between GSS and the others nomograms of 50% in time acquisition, with a power of 80% and a significance level of 0.05, based on the initial findings of the 20 cases studied for training the observer. With these parameters, we reached a total number of 18 patients. We studied 48 trying to improve the success comparison among the nomograms.

To check the normality, we used the Wilk—Shapiro test. GSS distribution was non-normal. So, to verify if the differences between the acquisition times were significant, we performed an analysis of variance (ANOVA) for repeated measures and compared these analyses to others performed with Wilcoxon signed-rank test, showing that they were similar, supporting our ANOVA use for this study. To check if they were all different, we did a multiple comparison by the Tukey method, comparing the scoring systems in pairs. We generated receiver operator characteristic (ROC) curves for each scoring system. We calculated the area under the curve and asymptotic 95% confidence intervals were calculated for each ROC curve. We performed all statistical analyses using SPSS 19.0 software for Windows (SPSS Inc., Chicago, USA). A p value of <0.05 was considered statistically significant.

## RESULTS

### Perioperative data

Demographic data are shown in the Table-1. The immediate success rate was 66.7% (29.2% of stone free and 92.4% for final success rate after a mean of 1.29 auxiliary procedures), and complications occurred in 16.7% of cases. The average operative time was122 minutes (Table-1).

**Table 1:** Baseline characteristic of study patients.

No. pts		48
Mean ± SD age		46.4±14.3
% Male		29.16
Mean ± SD body mass index (kg/m2)		28.3±7.6
% Right kidney		56.3
**% American Society of Anesthesiologist score:**		
	I / II / III / IV	50 / 43.7 /6.3 / 0
	% Ipsilateral Prior surgery(PCNL or SWL)	25
	% of patients with spina bifida or spine injury	0
	% Immediate success (fragments ≤4mm POD1)	66.7
% Stone-free (no fragments POD1)		29.2
%Final Success Rate (after all auxiliary procedures)		92.3
% Complications		16.7
Mean ± SD operative time (min)		122.3±46.1
**% Guy´s Stone Score:**		
	1	10.4
	2	39.5
	3	33.4
	4	16.7
**% S.T.O.N.E. Nephrolithometry**		
	5-6	18.75
	7-8	50
	9-12	31.25
**% CROES Nephrolithometric Nomogram**		
	130-169	16.75
	170-219	39.5
	>220	43.75

### Nomograms application speed

**Figure 1:**
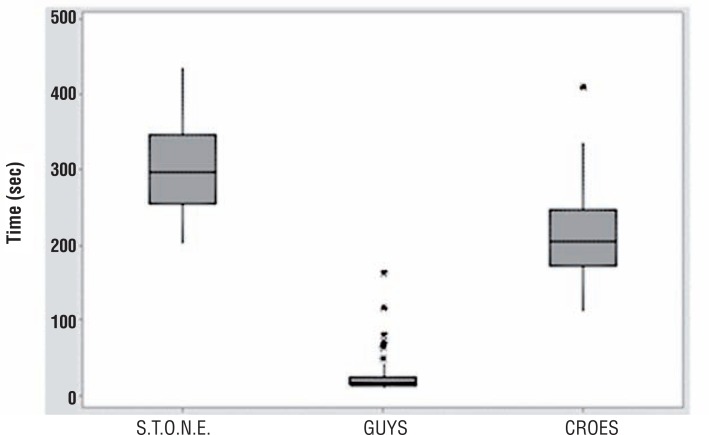
Time of application of the nomograms (in seconds).

Mean application time for the GSS was 27.5±30.0 seconds, significantly shorter than the 300.6±56.5 seconds for STONE and 213.4±59.4 seconds for CROES. There was a significant difference between all groups (p <0.001) (Figure-1).

### Scoring Systems Reliability

**Figure 2:**
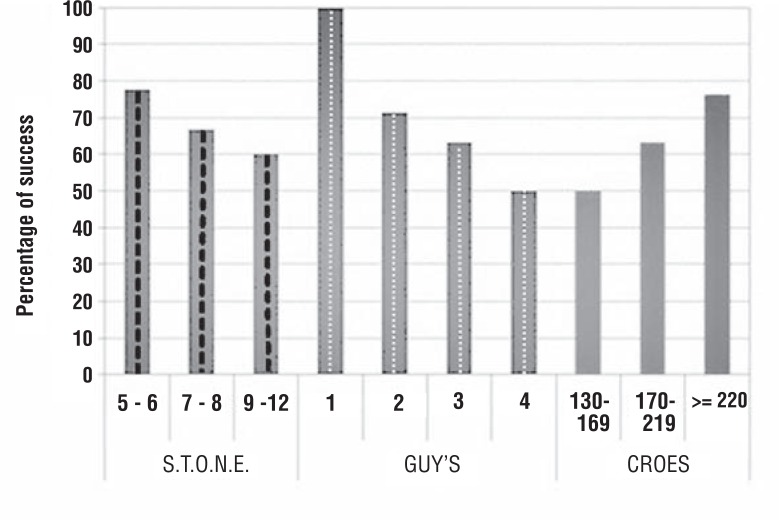
Percentage of success by groups of scores.

**Figure 3:**
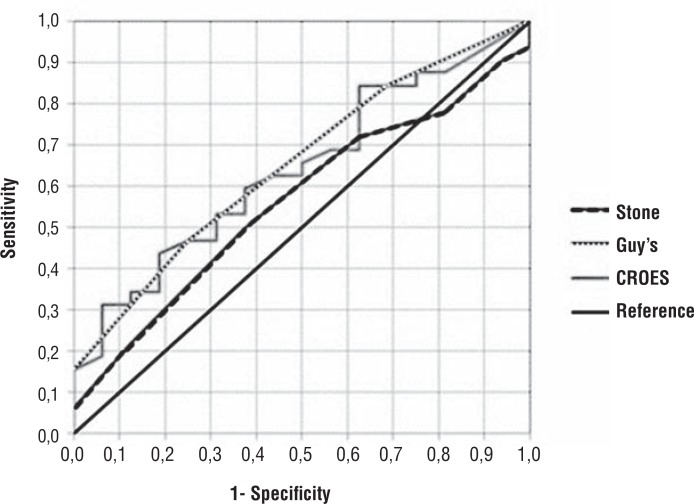
ROC curve for STONE score, Guy's score, CROES nomogram and reference.

After the two by two comparison of the AUC, there was no significant difference among the GSS (AUC=0.653), STONE (AUC=0.563) and CROES (AUC=0.641) in the capacity to predict immediate success of PCNL (STONE x GSS: p= .445; STONE x CROES: p=0.513; GSS x CROES: p=0.912). Figure-2: shows the percentage of success by groups of scores. Figure-3: shows the AUC and ROC curves for each of the scoring systems. All scoring systems demonstrated similar accuracy.

## DISCUSSION

Instruments that aim to classify the surgical risk and estimate the percentage of success involve risk scales, nomograms, probability Tables and analysis by regression trees. They are very useful because they help the surgeon in planning surgical strategies, result in better counselling of the patient and allow the comparison of outcomes between the experiences of different institutions.

The three nomograms evaluated in this study, GSS (8), STONE (10) and CROES (9) have been recently proposed as tools to predict success in PCNL. They use measurable and qualitative parameters, acquired from preoperative imaging studies and medical history. Despite the heterogeneity between nomograms, the three aim to classify patients into groups with different graduations of success in PCNL.

We have demonstrated that the nomograms were not significantly different in regards to the ability to predict success from PCNL. In the ROC curve analysis for the three scoring systems, we found that the Area Under the Curve (AUC) of CROES and GSS were similar (0.641 and 0.653, respectively), while the STONE was lower (0.563). However, the two by two comparison between them revealed no significant difference (STONE x GSS: p=0.445; STONE x CROES: p=0.513; GSS x CROES: p=0.912). Other studies have compared the three nomograms and the results are similar to ours. In a study involving 246 patients, Labadie and Okhunov (19) found the area under the curve of GSS, CROES and STONE were 0.634; 0.671 and 0.670, respectively and also demonstrated that the nomograms were not significantly different. Noureldin at al. (20) also showed similar AUC between GSS and STONE. Sfoungaristos et al. (21) could demonstrate the correlation between higher complexity according to the three nomograms and use of fluoroscopy. It is important to note that the value of the AUC is relatively low, suggesting a low capacity of predicting success. In our point of view, the use of AUC actually does not reflect the real benefit of using any of these nomograms. When we evaluate the different groups created by the nomograms, we can clearly see that these groups are different among them regarding peri and postoperative data, according previous reports (10, 11, 13–15, 18). The information obtained is critical when counselling the patient about the expectations for the surgery. Moreover, in a group of urologists used to the nomograms, when one says that is going to operate a patient with a GSS 4, everyone knows the difficulties that are expected, including the anesthesiologist and staff room, facilitating the operative planning. This is exactly what we observe in our institution, where we have been using the GSS for the last years. Similar effect can be expected if the team is used to another nomogram.

Withington et al. (22) in a recent systematic review of the literature could not firmly recommend one nomogram over the others, but found that the quality of evidence supporting validation of the GSS was marginally superior. If all scoring system are good for predicting outcomes and are similar among then in their capacity of doing that, which one should be used in a daily basis? The applicability of a nomogram depends on how easy it is to be used in a clinical setting. Hence, time used for calculating the score is an important factor when considering routine use. For that reason, we decided to study the application time for each nomogram. To the best of our knowledge, this is the first study to address this issue. In our study, we could verify that the GSS, a visual method that requires no measurement, was the fastest to be calculated. There was a large difference between application times of scoring systems, and GSS was the fastest, with an average of 27.5 seconds followed by CROES with 213.4 seconds and STONE with 300.6 seconds (p<0.0001). With these findings, it has been demonstrated that the GSS could became the most practical nomogram to be used for predicting outcomes for PCNL. This new information could be useful for urologists who want to start using a nomogram but are unsure which one to choose. If all nomograms have similar ability to predict success, then choosing the quickest seems logical.

Our study is not without limitations. Being retrospective is a weakness, but as we evaluated basically CT scans and the clinical data was prospectively acquired, we believe this characteristic does not compromise the results. The number of patients is relatively small compared to other recent larger multicenter series, but the results related to predicting success were similar to the others studies and the analyzed cohort was large enough to identify statistically significant differences between the nomograms in regards to the acquisition time. In addition, we performed a standardized preand postoperative evaluation of all patients with CT scan, increasing the reliability of the outcome assessment. Only one observer did all the measures, what could cause some bias. However, this observer was previously trained by two experienced urologists and as we had a high concordance index among then, we believe that one evaluator would be adequate. All patients underwent surgery performed by the same experienced surgeon using the same technique, reducing the potential biases in PCNL outcomes. Finally, the success evaluation was very early and rigorous, but we believe this was the better moment to have adequate and standardized evaluation for all patients. Certainly, this early evaluation causes a relatively low success rate, however as our final success rate after all secondary procedures was 92.3% the difference among the groups created by the nomograms probably would not be significant. Considering this, we believed that the immediate success evaluation with CT scan would provide the best information for comparison.

In our study, we found that all three nomograms showed similar ability to predict success of PCNL but the GSS was quicker to use than the others. Maybe these nomograms can be automated, making them easier to use, but at present this is not available. The relative low AUC of the three nomograms calls attention for necessity of continuing development and improvement of these tools.

## CONCLUSIONS

All three nomograms showed similar ability to predict success of PCNL, however the GSS was the quickest to be applied, what is an important issue for routine clinical use when counseling patients who are candidates to PCNL.
